# Impact of sheep wool residues as soil amendments on olive beneficial symbionts and bacterial diversity

**DOI:** 10.1186/s40643-022-00534-2

**Published:** 2022-04-21

**Authors:** Michela Palla, Alessandra Turrini, Caterina Cristani, Laura Bonora, David Pellegrini, Jacopo Primicerio, Arianna Grassi, Filip Hilaj, Manuela Giovannetti, Monica Agnolucci

**Affiliations:** 1 Department of Agriculture, Food and Environment, University of Pisa, Via del Borghetto 80, 56124 Pisa, Italy; 2 National Research Council-Institute of BioEconomy (CNR-IBE), via Madonna del Piano 10, 50019 Sesto Fiorentino (FI), Italy

**Keywords:** Soil bacterial communities, Arbuscular mycorrhizal symbionts, PCR-denaturating gradient gel electrophoresis (PCR-DGGE), Mycorrhizal inoculum potential (MIP) bioassay, Agro-industrial residues valorization

## Abstract

In recent years the use of organic matter soil amendments, such as agricultural by-products, has been implemented with the aim of increasing soil fertility, while minimizing the environmental impact of agriculture. Sheep wool residues (SWR) have shown beneficial effects on plant nutrition and soil properties, while only few works assessed their impact on soil microbial communities. The main aim of this work was to investigate the possible valorization of two SWR types (scoured residues, white wool, WW, and carbonized scoured residues, black wool, BW) as organic soil amendments, in pot-grown olive trees, by evaluating their impact on soil bacterial communities and mycorrhizal symbionts. The two SWR types did not negatively impact on the diversity and composition of soil bacterial communities, as revealed by PCR-denaturating gradient gel electrophoresis (PCR-DGGE) of partial 16S rRNA gene, and on the activity of native arbuscular mycorrhizal fungi (AMF), while positively affecting plant growth. Only the highest doses of one SWR type (2% BW) caused a decrease in bacterial diversity and native AMF ability to colonize olive roots. DGGE bands sequencing allowed the identification of the major bacterial taxa. Sequences corresponding to *Ohtaekwangia* spp., Beta proteobacterium, *Blastocatella* sp., *Ramlibacter monticola* and *Massilia frigida/rubra*, *Dongia* sp. and Chloroflexi were mainly represented in SWR-amended soils, while those represented by *Chryseolinea soli* and Acidobacteria were abundant in control soil. Overall, this work showed that SWR may be valorized as organic soil amendments, as soil bacteria and AMF, representing key factors of biological soil fertility, were not negatively affected, while the activity of bacterial genera and species known for their ability to decompose complex compounds was boosted. Further studies will investigate the biodegradation efficiency of the diverse bacterial taxa developing in SWR-amended soils.

**Graphic Abstract:**

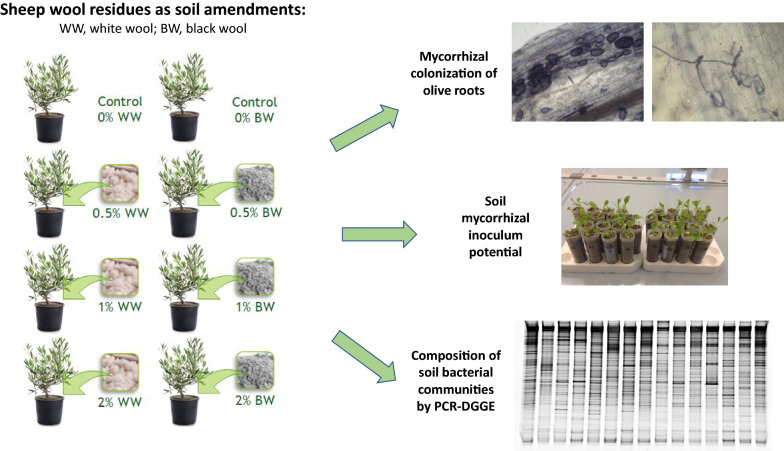

## Introduction

In recent years agricultural practices aimed at increasing soil fertility and carbon sequestration, and decreasing greenhouse gases emissions have been recommended, together with the reduction of mineral fertilizers and pesticides (IPCC 2018). Among the different practices, the use of organic matter soil amendments, such as agricultural by-products or wastes, has been implemented, leading to increases in crop production while minimizing the environmental impact of agriculture (Oldfield et al. 2019). In Europe, more than 200,000 tons of coarse wool are produced annually, of which about 18,000–20,000 tons in Italy (Zoccola et al. 2015). This type of wool, that does not meet the quality standards requested by the clothing industry, is classified as special waste by the European Commission, and it is often disposed of in landfill sites (Bhavsar et al. 2016).

However, several studies have been carried out on the utilization of sheep wool waste or wool residues in agriculture, aimed at testing their beneficial effects on soil properties and crop productivity, as well as improving the environmental sustainability of sheep wool supply chain. Actually, both wool residues, wood pellets and hydrolyzed wool waste were reported to stimulate plant growth and nutrition in different plant species, such as basil, thorn apple, broccoli, cluster bean, garden sage, maize, marigold, peppermint, ryegrass, sunflower, tomato, valerian, wheat and Swiss chard (Zheljazkov 2005; Nustorova et al. 2006; Zheljazkov et al. 2008, 2009; Gogos et al. 2013; Suruchi et al. 2014; Ordiales et al. 2016; Abdallah et al. 2019b). Moreover, recent studies reported that wool residues were able to enhance the physical and hydraulic properties of soils, by absorbing and retaining moisture, reducing soil bulk density and increasing total porosity and aggregate stability (Zoccola et al. 2015; Abdallah et al. 2019a).

Only few works assessed the impact of wool residues on soil microbial communities, species richness and composition, although specific microbiological studies have been performed with the aim of isolating wool-degrading bacteria, actinomycetes and fungi for their possible use as producers of keratinolytic enzymes (Korniłłowicz-Kowalska and Bohacz 2011; Petek and Logar 2021). Utilizing conventional microbiological analyses, increases in the number of soil bacteria and soil microbial biomass were detected after wool residues amendments, indicating their active participation in wool degradation (Nustorova et al. 2006; Zheljazkov et al. 2008). Potential detrimental effects of waste wool on biological soil fertility were assessed on an important group of beneficial fungi, arbuscular mycorrhizal (AM) fungi (AMF), which establish mutualistic symbioses with the roots of most crop plants. Unfortunately, the few available data do not support clear-cut conclusions, as the responses were not consistent across plant species (Zheljazkov 2005; Zheljazkov et al. 2008).

The aim of this work was to investigate the possible valorization of sheep wool residues (SWR) as organic soil amendments, in pot-grown olive trees, by evaluating their impact on soil bacterial and AMF communities. To this aim, we used two types of sheep wool residues (SWR) (scoured residues, white wool, WW, and carbonized scoured residues, black wool, BW) at 4 different SWR/soil ratios (w:w) (0, 0.5, 1.0, 2.0%). We determined: (i) the diversity and composition of soil bacterial communities by a culture-independent method, PCR-denaturating gradient gel electrophoresis (PCR-DGGE) of partial 16S rRNA gene; (ii) the activity of native AMF in the soil by the mycorrhizal inoculum potential (MIP) bioassay; (iii) the colonization of olive roots by native AMF after root clearing and staining.

## Material and methods

### Plant material and soil characteristics

The olive plants (*Olea europaea* L.) were obtained from Leccino cultivar, by rooting plant cuttings propagation method, to ensure the production of uniform plant material.

Twenty-centimetre-long branch portions were cut from the tip of 1-year-old healthy olive branches; each branch was cut 0.3 cm below a leaf node. The olive cuttings were placed in container deep enough to support the development of new roots, with the cut end buried in premoistened planting-medium by 2.5–3.8 cm. About 2 months after rooting process, the olive rooted cuttings were put in individual 10-cm-diameter nursery pots filled with a mix of washed sand and milled peat (1:1, v:v). Successively, rooted olive cuttings were transplanted into 0.35-L pots filled with the same potting medium, under lightly shaded conditions. In winter 2017, uniform olive plants with a single main stem were transplanted into pots containing 3.6 L of soil amended with increasing concentrations (0, 0.5, 1.0, 2.0%) of two different types of sheep wool residues (SWR), as described below.

The soil used was collected from the topsoil layer (0–30 cm) of an olive orchard at the experimental site of the Italian National Research Council (CNR) located in Follonica (southern Tuscany, “Santa Paolina” experimental farm (Lat. 43° 49´ 3.032" N, Long. 11° 12´ 4.858" E; 12 m a.s.l.). Physical and chemical characteristics of the soil were as follows: sand 75.63%; silt 8.63%; clay 15.74% (texture ranging from clay loam to sandy loam, Soil Survey Division Staff, 2017); organic matter 0.75%; pH 6.68; total nitrogen 0.08%; available phosphorus 9.9 mg/kg; exchangeable potassium 232 mg/kg; electrical conductivity (EC) 0.705 mS/cm, cation exchange capacity (CEC) 5.6 meq/100 g, active carbonates < 0.1%.

### Sheep wool residues (SWR) treatments

The SWR were acquired from the wool scouring company Carbon S.r.l., located in Vernio (Tuscany, Italy). Two SWR types, resulting from two different stages of the wool processing chain (regulated by the Commission Regulation—EU 1063/2012) were utilized: (1) white wool residue (WW), obtained from the mechanic beating of scoured wool and consisting of wool fibre and vegetal residues; (2) black wool residue (BW), obtained from the “carbonization” of the scoured and beaten wool with a solution of sulfuric acid. To determine carbon (C), nitrogen (N), hydrogen (H) and sulphur (S) contents, dry samples of wool residues (WW and BW) were analysed using a CHN Elemental Analyzer (Carlo Erba Instruments, mod 1500 series 2). Percentage contents of N, C, H and S were almost similar in WW and BW: N 11.62, C 44.09, H 6.84, S 2.72 and N 12.31, C 41.87, H 6.35, S 5.76, respectively.

### Experimental design

To ensure that the culture substrate was physically homogeneous throughout the pots, the soil was prepared using an electric concrete mixer (100-L capacity, 0.4 hp, 23 rpm). Both control soil and soil–wool mixtures had 20 min of mixing inside the mortar. WW and BW soil–wool mixture samples with (0, 0.5, 1 and 2%) (w:w) were prepared, resulting in 7 different culture substrates, with three replicates for each treatment. The experiment was carried out outdoor from April 2017 to February 2020, at the Institute of BioEconomy (IBE) of CNR in Florence (Central Italy, Lat. 43°49′3.032" N and Long. 11°12′4.858′′ E, 40.5 mt. above s.l.). Pots were arranged in a completely randomized distribution, plants were organized with a fixed-position arrangement (North–South exposition) and placed in open field.

Climatic conditions were typical for Mediterranean regions, where, on average, there are 88 days per year with more than 0.1 mm of rainfall, the driest weather is in July (an average of 39.6 mm), while the wettest weather is in November (an average of 111.2 mm). July is the hottest month with a mean temperature of 24 °C, while January is the coldest having an average of 6 °C. No fertilization and agronomic treatments were supplied during all the experimentation time. The plants were well watered through the experiment thanks to a drip irrigation system with 300 mL each day during summer season. This solution allowed the supply of a regular and uniform water quantity, enough to cover the 80–100% of the field capacity.

Collar diameters of olive plants were measured in six subsequent dates and analysed by one-way ANOVA. The occurrence of significant differences among treatments was established performing the LSD post hoc test. The statistical analyses were carried out in IBM SPSS statistics version 24 software (IBM Corporation, Armonk, NY, USA).

From each pot, two soil cores of 3 cm diameter were collected, taking care to collect also the roots of the olive trees. The two soil subsamples were subsequently mixed in order to produce a single sample.

### Diversity and composition of soil bacterial communities by PCR-DGGE

#### DNA extraction from soil samples and PCR amplification

Genomic DNA was extracted from 250 mg soil samples using DNeasy® PowerSoil Kit® (QIAGEN Group, Germantown, MD) according to the manufacturer’s protocol. The extracted DNA was stored at − 20 °C and subsequently used for the analysis of soil bacterial communities. The amplification of the variable region V3–V5 of 16S rDNA was carried out using the primers 341F (5’-CCT ACG GGA GGC AGC AG-3’) and 907R (5’-CCG TCA ATT CCT TTR AG TTT-3’) (Yu and Morrison 2004). At its 5’ end, the primer 341F had an additional 40-nucleotide GC-rich tail (5’-CGC CCG CCG CGC CCC GCG CCC GTC CCG CCG CCC CCG CCC G-3’). Amplification reaction was prepared in a final volume of 50 μL, using 10–20 ng of DNA, 5 μL of Ex Taq Buffer 10X (Takara Biotechnology), 1.25 U of Takara Ex Taq (Takara Biotechnology), 0.2 mM of each dNTP (Takara Biotechnology) and 0.5 μM of each primer (Eurofins). The fragment obtained was about 560 bp long. The reaction was carried out using an iCycler-iQ Multicolor Real-Time PCR Detection System (Bio-Rad) with the following denaturation, amplification and extension procedure: at 95 °C for 1′; at 94 °C for 30′′, at 60 °C for 30′′, at 72 °C for 30′′ (for 35 cycles); at 72 °C for 5′. The presence of amplicons was confirmed by electrophoresis in 1.5% (w/v) agarose gels stained with 20,000X REALSAFE Nucleic Acid Staining Solution (Durviz, s.l., Valencia, Spain). All gels were visualized using UV light and captured as TIFF format files using the UVI 1D v. 16.11a program for the FIRE READER V4 gel documentation system (Uvitec Cambridge, Eppendorf, Milan, Italy).

#### DGGE and profile analyses

For DGGE analyses, 20 μL of amplicons were separated in 8% (w/v) polyacrylamide gels with a 36–58% urea–formamide gradient, using the DCode™ Universal Mutation Detection System (Bio-Rad, Milan, Italy). Gels were run at 90 V and 60 °C for 16 h, stained for 30′ in 500 mL of TAE buffer 1X containing 50 μL of Sybr Gold Nucleic Acid Gel Stain (Thermo Fisher Scientific, Italia) and visualized as previously described. The sample V18 was added on each side and in the centre of DGGE gels as DGGE marker.

DGGE profiles were digitally processed with BioNumerics software version 7.6 (Applied Maths, St-Martens-Latem, Belgium) and bacterial community composition was assessed by cluster analysis of DGGE profiles, as reported in Palla et al. (2018). Similarities between DGGE patterns were calculated by determining Pearson’s similarity coefficients for the total number of lane patterns from the DGGE gel using the band-matching tool with an optimization of 1%. The similarity coefficients were then used to generate the dendrogram utilizing the clustering method UPGMA (unweighted pair group method using arithmetic average).

DGGE banding data were used to estimate four different indices treating each band as an individual operational taxonomic unit (OTU). Richness (*S*) indicates the number of OTUs present in a sample and was determined from the number of fragments. Shannon–Weaver (Hs) and the dominance index of Simpson (*D*) were calculated using the equations Hs =  −Σ(*P*_*i*_ x ln*P*_*i*_) and *D* = Σ*P*_*i*_^2^, respectively, where the relative importance of each OTU is *P*_i_ = *n*_i_*N*^−1^, and *n*_*i*_ is the peak intensity of a band and *N* is the sum of all peak intensities in a lane. Evenness index (*E*), which allows the identification of dominant OTUs, was calculated as *E* = *H* (lnS)^−1^.

One-way ANOVA was applied to diversity indices with SWR/soil ratios as the variability factor. The means were compared by the Tukey's test (*P* < 0.05). Analyses were carried out with the SPSS version 23 software (IBM Corp., Armonk, NY, USA).

#### DGGE band sequencing

The main bands of DGGE profiles were excised from the gels for sequencing at the Eurofins Genomics MWG Operon (Ebersberg, Germany) as reported in Agnolucci et al. (2019). DNA was extracted by eluting for 3 days in 50 μL UltraPure™ DNase/RNase-Free Distilled Water (Invitrogen) at 4 °C. Two microliters of the supernatant diluted 1:10 were used to re-amplify the V3–V5 region of the DNA, using the 341F primer without the GC clamp. PCR products were than purified with the QIAquick PCR Purification Kit (Qiagen) according to the manufacturer's protocol, quantified and 5’ sequenced by Eurofins Genomics (Ebersberg, Germany). Sequences were analysed using BLAST on the NCBI web (http://blast.ncbi.nlm.nih.gov/Blast.cgi). The related sequences were collected and aligned using MUSCLE (Edgar 2004a, b), and phylogenetic trees were constructed using the Neighbor-Joining method based on Kimura’s 2-parameter model (Kimura 1980) in Mega X software (http://www.megasoftware.net/) with 1000 bootstrap replicates. Sequences were submitted to the European Nucleotide Archive under the accession numbers: from OU745530 to OU745538 (WW) and from OU745381 to OU745389 (BW) (Project: PRJEB47775).

### Mycorrhizal inoculum potential of the soil

Mycorrhizal inoculum potential (MIP) bioassay was performed to verify the activity of native AMF occurring in the soil of pot-grown olive plants and was assessed using *Cichorium intybus* L. as host plant, as described in Turrini et al. (2018). Briefly, soil samples from each pot (50 g soil) were dried, sieved using a 4-mm sieve, and put in 50-mL tubes. For each MIP determination, six replicated tubes were used, filled with 45 mL of soil and sown using the biotest plant. Then, they were put in sun-transparent bags and maintained in a growth chamber at 27 °C under a 16/8 h light/dark daily cycle until harvest. Four days after germination, plants were thinned to three per tube. Each tube was watered as needed. Plants were harvested 28 days after sowing and shoots were excised and discarded. Roots were removed from soil, washed with tap water, then cleared in 10% KOH in a 80 °C water bath for 15′, neutralized in 2% aqueous HCl, and stained with 0.05% Trypan blue in lactic acid. The percentage of AMF colonization was calculated using a dissecting microscope at × 25 or × 40 magnification and the gridline intersect method (Giovannetti and Mosse, 1980).

### Mycorrhizal colonization

The percentage of AMF root colonization was determined on 5 g of thoroughly washed olive root samples, after clearing and staining, as described above. Percentages of AM fungal root colonization were assessed on representative root samples from each plant under a dissecting microscope (Wild, Leica, Milano, Italy) at × 25 or × 40 magnification by the gridline intersect method (Giovannetti and Mosse 1980).

Data of MIP and olive root mycorrhizal colonization (after arcsin transformation) were analysed by one-way ANOVA. The occurrence of significant differences among treatments was established performing the Tukey post hoc test. The statistical analyses were carried out in IBM SPSS statistics version 23 software (IBM Corporation, Armonk, NY, USA).

## Results

### Plant growth

**Fig. 1:**
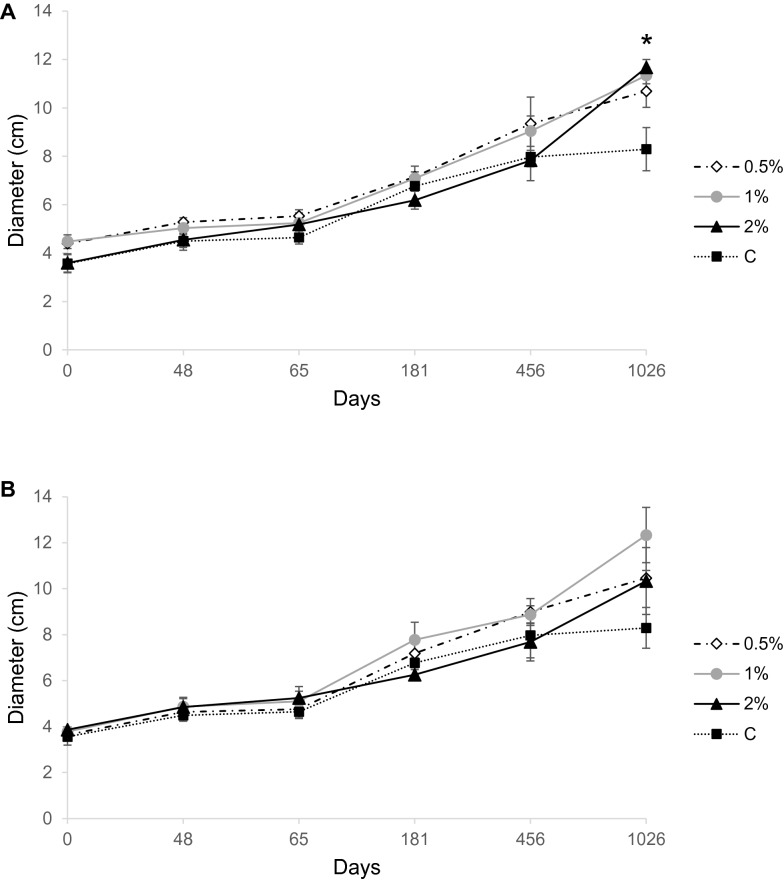
Shoot diameters (means ± standard errors of three replicates per treatment) of olive trees grown in pots for 1026 days and fertilized with two types of sheep wool residues (SWR). **A** white wool, WW, and **B** black wool, BW, used at 4 different SWR/soil percentages: 0 (C), 0.5, 1.0, 2.0%. The asterisk indicates least significant differences (LSD) between treatments and control (*P* < 0.05)

Here we report data on shoot diameter of olive plants grown for 1026 days in the experimental trial (Fig. 1). At the end of the experiment, olive treated with WW and BW amendments showed 33% mean growth increases, compared with the control. In particular, significant differences were found when olive plants were treated with WW (Fig. 1).

### Soil bacterial communities diversity

The DNA extracted from soil samples was successfully amplified using the primer pair 341 F-GC/907 R, obtaining a fragment of the expected size (ca. 560 bp), corresponding to the V3–V5 region of the 16S rRNA gene.

**Fig. 2:**
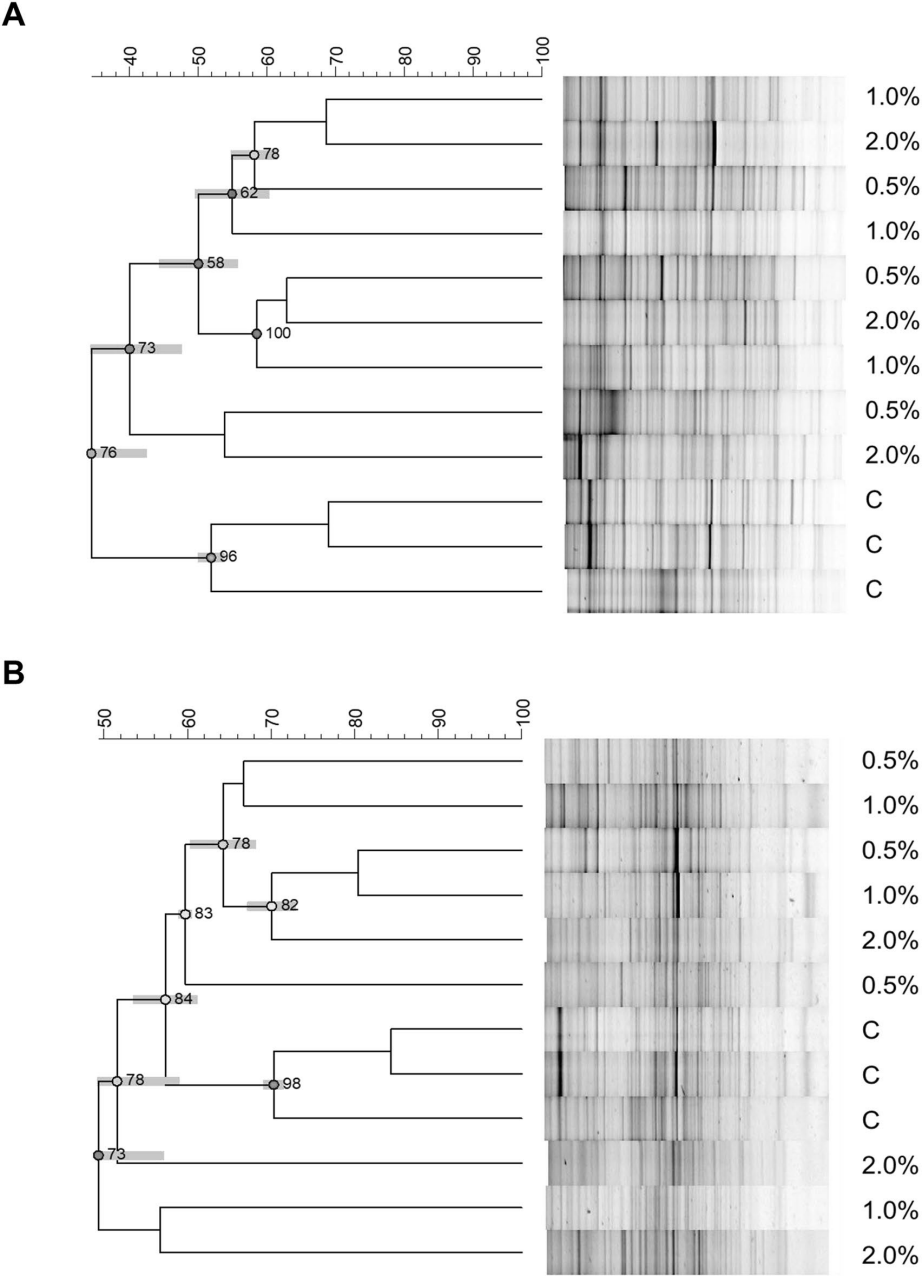
Cluster analyses of bacterial DGGE profiles. Dendrograms obtained by UPGMA (Unweighted Pair Group Method Using Arithmetic Average) analysis, using Pearson’s coefficient, based on bacterial DGGE profiles obtained from pot-grown olive trees fertilized with two types of sheep wool residues (SWR). **A** white wool, WW, and **B** black wool, BW, used at 4 different SWR/soil percentages: 0 (C), 0.5, 1.0, 2.0%. Cophenetic correlation is shown at each node by numbers and coloured dots, ranging between green–yellow–orange–red, according to decreasing values. Standard deviation is shown at each node by a grey bar

DGGE profiles of PCR products obtained from WW and BW-amended soils showed distinctive patterns, characterized by intense and clearly defined fragments. Soil bacterial community composition was assessed by cluster analysis of DGGE profiles (Fig. 2). The two dendrograms clearly separated the bacterial communities of control soils from those of soils amended with WW and BW, with a low similarity, 34% and 49–57%, respectively. In particular, as to WW, the first subcluster included all the bacteria of the amended soil samples, although no discrimination was evident across the different concentrations of WW amendments. As to BW it is interesting to note that two samples containing 2% wool clustered separately showing different bacterial communities.

DGGE profiles were also used to estimate S, Hs, D and Jp biodiversity indices. No statistically significant differences were found among the different SWR treatments for Hs, D and Jp, while the highest levels of BW (2%) significantly decreased the Richness index (S), which was lower than that of all the other treatments (Tables 1 and 2).

**Table 1:** Diversity indices calculated from bacterial DGGE profiles associated with pot-grown olive trees fertilized with sheep white wool residues WW, used at 4 different WW/soil percentages

	Treatment	Richness (S)	Simpson (D)	Shannon–Weaver (Hs)	Evenness (Jp)
WW	C	38 ± 1.00a*	0.04 ± 0.007a	3.37 ± 0.07a	0.93 ± 0.02a
	0.5%	37 ± 2.52a	0.04 ± 0.002a	3.34 ± 0.06a	0.93 ± 0.01a
	1.0%	39 ± 2.91a	0.04 ± 0.001a	3.38 ± 0.06a	0.93 ± 0.00a
	2.0%	35 ± 1.45a	0.06 ± 0.007a	3.15 ± 0.08a	0.89 ± 0.03a

*Values (means ± standard errors of three replicates per treatment) followed by the same letter in a column are not significantly different at *P* < 0.05 (Tukey’s post hoc test)

**Table 2:** Diversity indices calculated from bacterial DGGE profiles associated with pot-grown olive trees fertilized with sheep black wool residues, BW, used at 4 different BW/soil percentages

	Treatment	Richness (*S*)	Simpson (*D*)	Shannon–Weaver (Hs)	Evenness (Jp)
BW	C	39 ± 0.67a*	0.05 ± 0.005a	3.37 ± 0.04a	0.92 ± 0.01a
	0.5%	39 ± 0.88a	0.05 ± 0.006a	3.33 ± 0.06a	0.91 ± 0.02a
	1.0%	39 ± 0.67a	0.04 ± 0.008a	3.35 ± 0.10a	0.92 ± 0.02a
	2.0%	34 ± 1.53b	0.05 ± 0.003a	3.26 ± 0.05a	0.92 ± 0.00a

*Values (means ± standard errors of three replicates per treatment) followed by the same letter in a column are not significantly different at *P* < 0.05 (Tukey’s post hoc test)

**Fig. 3:**
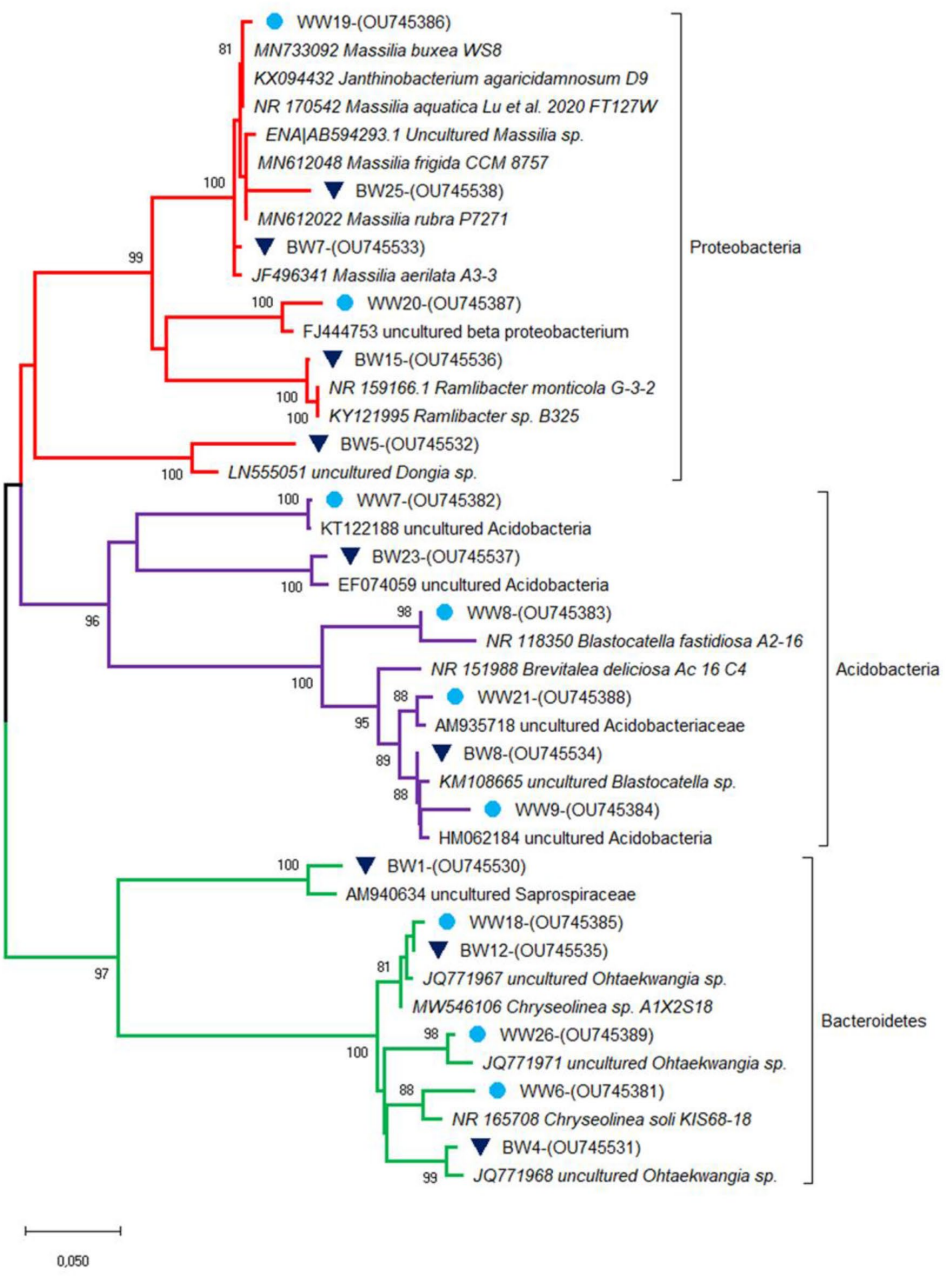
Affiliation of the main sequences retrieved from the predominant DGGE gel fragments with the existing sequences of V3–V5 region of 16S rRNA gene. Sequences belong to soil bacterial communities associated with pot-grown olive trees fertilized with two types of sheep wool residues (SWR) (white wool, WW and black wool, BW), used at 4 different SWR/soil percentages: 0 (C), 0.5, 1.0, 2.0%. Phylogenetic analysis was inferred by using the Maximum Likelihood method based on the Kimura 2-parameter model. Bootstrap (1000 replicates) values below 70 are not shown. Evolutionary analyses were conducted in MEGA X. The sequences from the database are indicated by their accession numbers. The DNA sequences retrieved in this work are indicated by their corresponding band number and their accession number

In order to identify the major bacterial taxa characterizing the experimental soils, bands of interest were excised from DGGE gels, sequenced and affiliated to genera and species by using BLAST and phylogenetic tree analyses. Sequences belonged to four phyla: Acidobacteria (*Blastocatella* spp., Acidobacteriaceae), Bacteroidetes (*Algoriphagus terrigena*, *Chryseolinea* spp., *Ohtaekwangia* spp., Saprospiraceae), Chloroflexi and Proteobacteria (*Dongia* sp., Beta proteobacterium, *Massilia* spp., *Ramlibacter monticola*) (Fig. 3 and Table 3).

**Table 3:** Identification of sequences retrieved from fragments of PCR-DGGE analysis

DGGE fragment	Identity (%)	Organism	Genebank accession number
C6	96.60	*Chryseolinea soli* strain KIS68-18	NR_165708
C7	99.29	Uncultured Acidobacteria clone TGRWLFZ-16 s-SSe277	KT122188.1
WW8	97.16	*Blastocatella fastidiosa* strain A2-16	NR_118350
WW9	96.00	Uncultured Acidobacteria	HM062184.1
WW18	98.96	Uncultured *Ohtaekwangia* sp. clone CS11	JQ771967
WW19	98.72	*Massilia buxea* strain WS8	MN733092.1
WW20	97.20	Uncultured Beta proteobacterium clone 4y-82	FJ444753.1
WW21	97.96	Uncultured Acidobacteriaceae	AM935718.1
WW25	95.21	Uncultured Acidobacteriaceae	HE860923.1
WW26	98.74	Uncultured *Ohtaekwangia* sp. clone CS15	JQ771971.1
WW27	94.22	Uncultured Acidobacteria bacterium clone KF4-062	JF521709.1
BW1	98.80	Uncultured Saprospiraceae clone A9-33	AM940634
BW3	97.78	*Algoriphagus terrigena* strain DS-44	NR_043616
BW4	97.60	Uncultured *Ohtaekwangia* sp. clone CS12	JQ771968.1
BW5	96.18	*Dongia* sp. clone SBROB5_53	LN555051
BW7	98.29	*Massilia aerilata* strain A3-3	JF496341
BW8	98.53	Uncultured *Blastocatella* sp. clone SBK39	KM108665.1
BW10	93.97	Beta proteobacterium Schreyahn AOB SSU Aster 2	AY795686.2
BW12	99.18	*Chryseolinea* sp. strain A1X2S18	MW546106
BW15	98.58	*Ramlibacter monticola* strain G-3-2	NR_159166
BW16	95.17	Uncultured *Chloroflexi*	FM253679
BW21	94.00	Uncultured Acidobacteria	JQ026742.1
BW23	97.98	Uncultured Acidobacteria clone GASP-WB2S3_E12	EF074059.1
BW25	96.60	*Massilia frigida* strain CCM 8757/*M. rubra* strain P7271	MN612048/MN612022

*C* untreated soil, *WW* white wool, *BW* black wool

Among them, sequences corresponding to *Ohtaekwangia* spp. (WW18, WW26), Beta proteobacterium (WW20), *Blastocatella* sp. (BW8), *Ramlibacter monticola* (BW15) and *Massilia frigida/rubra* (BW25), *Dongia* sp. (BW5) and Chloroflexi (BW16) were mainly represented in SWR-amended soils. Conversely, sequences represented by *Chryseolinea soli* (C6) and Acidobacteria (C7, BW23) were abundant in the untreated soil and showed a general decrease in the soil treated with SWR. Moreover, in BW-amended soils, the fragments corresponding to a Beta proteobacterium (BW10) and to an Acidobacteria (C7) showed a low intensity in soil samples treated with 2.0% BW residues (Fig. 3 and Table 3).

### AMF activity and colonization level

The activity of AMF propagules, evaluated by MIP bioassay, was not significantly affected by the different levels of SWR, as the percentage of colonized root length of the test host plant *C. intybus* ranged from 44.3% in control samples to 53.9 and 67.9% in WW and BW-amended soils, respectively (Tables 4 and 5). These data indicate that both WW and BW did not produce detrimental effects on the mycorrhizal potential of the SWR-amended soils.

**Table 4:** Soil mycorrhizal inoculum potential (MIP) and mycorrhizal colonization of pot-grown olive trees fertilized with sheep white wool (WW) residues, used at 4 different WW/soil percentages

	Treatment	MIP (%)	Mycorrhizal colonization (%)
WW	C	44.3 ± 5.8a*	83.3 ± 3.3a
	0.5%	53.9 ± 9.5a	80.0 ± 5.8a
	1.0%	50.4 ± 5.8a	76.7 ± 6.7a
	2.0%	51.4 ± 4.0a	80.0 ± 5.8a

*Values (means ± standard errors of three replicates per treatment) followed by the same letter in a column are not significantly different at *P* < 0.05 (Tukey’s post hoc test)

**Table 5:** Soil mycorrhizal inoculum potential (MIP) of pot-grown olive trees fertilized with sheep black wool (BW) residues, used at 4 different BW/soil percentages

	Treatment	MIP (%)	Mycorrhizal colonization (%)
BW	C	44.3 ± 5.8a*	83.3 ± 3.3a
	0.5%	61.0 ± 8.5a	80.0 ± 5.8a
	1.0%	67.9 ± 10.6a	76.7 ± 3.3a
	2.0%	44.3 ± 6.6a	53.3 ± 3.3b

*Values (means ± standard errors of three replicates per treatment) followed by the same letter in a column are not significantly different at *P* < 0.05 (Tukey’s post hoc test)

Mycorrhizal colonization of olive roots was not affected by WW treatments, as the percentage of colonized root length varied from 76.7 to 83.3% (Table 4). However, mycorrhizal colonization of olive roots drastically decreased (by 36%) in plants grown in soils amended with 2% BW, showing that high doses of BW may hinder the development of the beneficial AMF symbiosis (Table 5).

## Discussion

This work showed that sheep wool residues may be valorized as organic soil amendments in olive trees, as they did not negatively impact on the diversity and composition of soil bacterial communities and on the activity of native AMF, while positively affecting plant growth. Only the highest doses of one SWR type (2% BW) caused a decrease in bacterial diversity and native AMF ability to colonize olive roots.

### Plant growth

Improved plant growth is generally regarded as one of the benefits provided by SWR soil amendments. Here, olive shoot diameters were significantly influenced by WW, although growth responses depended on the concentration. Our results are in agreement with those obtained with different plant species, such as basil, thorn apple, broccoli, cluster bean, garden sage, maize, marigold, peppermint, ryegrass, sunflower, tomato, valerian, wheat and Swiss chard (Zheljazkov 2005; Nustorova et al. 2006; Zheljazkov et al. 2008, 2009; Gogos et al. 2013; Suruchi et al. 2014; Ordiales et al. 2016; Abdallah et al. 2019b). Such effects have been ascribed to the protein keratin, the main component of sheep wool, whose slow degradation in the soil releases, beyond carbon (ca. 50%), plant mineral nutrients, first of all nitrogen (16–17%) and sulphur (2–4%), and also Ca, Na, K, P, Mg, Zn, Fe, Cu, and Mn (Verville 1996; Zheljazkov et al. 2009; Petek and Logar 2021). Indeed, other components may contribute to plant growth promotion, as raw wool may contain up to 40% of impurities, including lanolin, dirty soil, grease and vegetable matter (Petek and Logar 2021).

### Soil bacterial communities diversity as affected by SWR treatments

Cluster analysis of DGGE profiles detected significantly different bacterial community profiles in soils treated with WW and BW, compared with those of the untreated control soil, indicating that SWR amendments did not negatively affect soil microbiota and that, as other types of organic matter, were able to boost the activity of microorganisms, primary decomposers of organic matter (Ishii et al. 2000; Schloss et al. 2003; Fließbach et al. 2007). This is in agreement with previous data on soil wool-waste amendments, which induced a shift in the structure of soil microbial community, as assessed by fatty acid methyl esters (FAMEs) analyses, and increased microbial biomass in the soil of pot-grown marigold plants (Zheljazkov et al. 2008).

As to the different SWR doses, in addition to the shift in the bacterial community composition, also its richness was decreased by 2% BW soil amendments, compared with the untreated control soil, showing that such a concentration should be avoided in order to maintain high bacterial diversity.

The sequencing of DGGE bands detected 8 taxa assigned to uncultured members of the Acidobacteriaceae (Phylum Acidobacteria) both in SWR-treated and control soils, confirming their widespread occurrence in soil environments. Members of the Acidobacteriaceae family are metabolically diverse and several strains have been shown to be able to degrade cellulose, chitin, starch and xylan (Huber et al. 2015). Thus, it is conceivable that they are active in control agricultural soil, where plant and insect residues occur, and also in SWR-amended soils, where wool residues may represent a rich source of degradable compounds.

*Massilia frigida/rubra* was mostly retrieved in both BW and WW-treated soils, consistent with their isolation from highly diverse environments, including soil, rhizosphere and endorhizosphere (Ofek et al. 2012), while culture-independent methods detected *Massilia* sequences also in fungal structures (Agnolucci et al. 2015). Some *Massilia* strains have been shown to possess Plant Growth Promoting (PGP) activities, being able to produce antibiotics, siderophores and indole-acetic acid, while others were reported to hydrolyze animal proteins like gelatin and to degrade cellulose and starch (Ofek et al. 2012; Raths et al. 2020), and to degrade organic chemicals, such as benzene, toluene, ethylbenzene and xylene (Son et al. 2021). Accordingly, the detection of *Massilia* sequences in our SWR-amended soils, rich in organic matter, is compatible with the organic matter degradation activity of some strains.

Sequences of two betaproteobacteria were mainly found in WW and BW, consistent with their occurrence in different habitats. Members of Betaproteobacteria showed a large range of metabolic and ecological characteristics, encompassing bacterial isolates able to degrade complex aromatic hydrocarbons, together with chloroaromatic, nitroaromatic, aminoaromatic compounds and kraft lignin (Ramana et al. 2006; Shi et al. 2013; Tan and Parales 2019) and to establish mutualistic symbioses with plants, such as legumes, fungi and animals (Degli Esposti et al. 2018).

Within Bacteroidetes, the genus *Ohtaekwangia*, belonging to the family Fulvivirgaceae within the order Cytophagales, was reported to occur in marine sand and also in the rhizosphere of cucumber, sunflower, maize, soybean and turfgrass (Yoon et al. 2011; Zhang et al. 2011; Gaggia et al. 2013; Tejeda-Agredano et al. 2013; Tian and Gao 2014; Correa-Galeote et al. 2016), where it probably plays a role in the degradation of cellulosic root-derived matter. Interestingly, during oil mill waste composting, when sheep manure was used as bulking agent, the relative number of *Ohtaekwangia* sequences increased across the maturing phases, detecting this genus as a biomarker of compost maturation (Tortosa et al. 2017). Accordingly, other authors found that *Ohtaekwangia* was one of the core genera in mature granules of an aerobic granular sludge wastewater treatment plant (Świątczak and Cydzik-Kwiatkowska 2018). The occurrence of highly intense bands of this genus in the experimental soils amended with the two different types of SWR compared with control soils may be indicative of *Ohtaekwangia* positive role in the decomposition of sheep wool, which mainly consists of organic matter, such as keratin, lanolin, grease and vegetable residues (Petek and Logar 2021).

Interestingly, a sequence assigned to an uncultured Saprospiraceae prevailed in BW, consistent with previous findings on members of the family, that showed the ability to degrade complex organic compounds in the environment and in activated sludge wastewater treatment systems (McIlroy and Nielsen 2014).

Only one band whose sequences were ascribed to *Dongia* sp. and Chloroflexi were retrieved from BW. Their occurrence is not surprising, as, despite their occurrence in a wide variety of environments, they were detected in activated sludge plants, where they may play a key role in the degradation of complex organic compounds (Liu et al. 2010; Baik et al. 2013; Kim et al. 2016; Speirs et al. 2019).

Interestingly, *Chryseolinea soli* was mainly retrieved from control soils. The genus *Chryseolinea* (Cytophagales, Bacteroidetes) has been established in 2013 and consists of three described species isolated from soil in Germany, Korea and China (Kim et al. 2013; Wang et al. 2018; Lee et al. 2019). The isolates of the three species have diverse phenotypic characteristics in common, such as the inability to degrade cellulose, but no peculiar traits have been detected so far, allowing speculations other than a specific inhibiting activity of SWR on this particular bacterial species.

The bacteria occurring in our SWR-amended soils have not been described as keratinolytic microorganisms, as no assessment of keratinolytic proteases is usually carried out in the description of new species. Though it has long been known that microbial keratinolytic proteases have a broad substrate range, being capable of hydrolyzing diverse proteins—beyond keratin—including casein, gelatin and collagen (Brandelli et al. 2010), that are utilized by most of the bacteria detected in this work.

### AMF activity and colonization ability

This work reports for the first time that soil mycorrhizal potential, as assessed by MIP bioassay, was not significantly affected by WW and BW treatments, independent of the dose. Although in BW-amended soils MIP values showed some differences, they did not statistically differ, due to replicate variability. Overall, SWR did not produce any negative effect on the activity of native AMF soil propagules, which maintained their ability to establish mycorrhizal symbioses in the test plant *C. intybus*. Our data are in agreement with previous findings on soil wool-waste addition in the field, which did not affect total numbers of AMF spores (Zheljazkov et al. 2008).

The finding that mycorrhizal colonization of olive roots was not affected by WW treatments supports previous data on basil plants treated with unprocessed and unwashed wool waste, while contrasting with data on thorn apple, where the percentage of mycorrhizal root length decreased even at low levels of the same type of wool amendments (Zheljazkov 2005). A similar detrimental effect on the development of AMF symbiosis was here detected in olive plants treated with the highest doses of BW (2%), which significantly reduced mycorrhizal colonization. Such high BW doses differentially affected the ability of AMF propagules, spores and extraradical mycelium, to colonize *C. intybus*, utilized as test plant in the MIP bioassay, and olive roots. This finding may be ascribed to wool decomposition processes effected by soil microorganisms during the experimental period that may originate degradation metabolites, hindering the ability of specific native AMF to form infection structures and colonize olive roots. Indeed, many diverse microorganisms, both non-keratinophilic and keratinophilic, dominate the different phases of wool decomposition in soil, producing enzymes and secondary metabolites able to inhibit the growth of several microbial taxa (Ghawana et al. 1997; Nigam and Kushwaha 1990). The higher mycorrhizal colonization levels detected by MIP bioassay may be due to the complete or partial disappearance of such labile compounds after 1026 days, as reported also for ryegrass seed germination (Nustorova et al. 2006).

## Conclusions

Present results met the objective of our study, which was carried out in order to valorize sheep wool residues (SWR) as organic soil amendments, by evaluating their impact on soil bacterial and AMF communities in pot-grown olive trees. The two SWR types positively affected olive growth, and did not negatively impact on the diversity and composition of soil bacterial communities. Neither the two SWR types nor their different doses applied to soil affected the activity of native AMF, as assessed by the MIP bioassay, while mycorrhizal colonization was decreased by 2% BW soil amendments. Our data on soil bacteria and AMF, that represent key factors of biological soil fertility, complement previous findings on the beneficial effect of SWR on soil properties, such as moisture retention, reduction of soil bulk density and increase of total porosity and aggregate stability (Zoccola et al. 2015; Abdallah et al. 2019b).

SWR treatments produced a shift in the composition of soil bacterial communities, boosting the activity of genera and species known for their ability to decompose complex compounds. However, the different microbial taxa active in SWR degradation were not isolated and characterized, as our work was based on molecular, culture-independent analysis. Further studies are needed to enhance our understanding of the biodegradation efficiency of the diverse bacterial genera and species developing in SWR-amended soils and to detect wool degradation products.

## Data Availability

All data generated or analysed during this study are included in this published article.

## Abbreviations

AMF: Arbuscular mycorrhizal fungi
BW: Black wool
CEC: Cation exchange capacity
D: Simpson
EC: Electrical conductivity
FAMEs: Fatty acid methyl esters
Hs: Shannon–Weaver
Jp: Evenness
MIP: Mycorrhizal inoculum potential
PCR-DGGE: PCR-denaturing gradient gel electrophoresis
PGP: Plant growth promoting
S: Richness
SWR: Sheep wool residues
UPGMA: Unweighted pair group method using arithmetic average
WW: White wool
